# Equity in training load: research design considerations for intervention assessment in sports science and physical therapy

**DOI:** 10.1007/s00421-025-05844-9

**Published:** 2025-06-14

**Authors:** Tomasz Kowalski, Moritz Schumann, Sebastian Klich, Michele Zanini

**Affiliations:** 1 Department of Physiology, Institute of Sport—National Research Institute, Warsaw, Poland; 2 Department of Sports Medicine and Exercise Therapy, Chemnitz University of Technology, Chemnitz, Germany; 3 Faculty of Physical Education and Sport, Wroclaw University of Health and Sport Sciences, Wroclaw, Poland; 4 School of Sport, Exercise, and Health Sciences, Loughborough University, Loughborough, UK; 5 School of Education, Childhood, Youth and Sport, The Open University, Milton Keynes, UK

**Keywords:** Training load, Study design, Research methodology, Training, Physical therapy, Exercise physiology

## Abstract

Training load (TL) assessment and management are important in athletic training, physical therapy, and sports science, as exercise-induced responses to the exercise stimulus depend on the dose of the applied exercise stimulus. Different TL may result in distinct adaptation, intervention feasibility, or injury risks, amongst other considerations. However, often studies are based on interventions with different TL between the groups, possibly influencing the research outcomes. This opinion paper discusses scenarios where TL matching may be beneficial, and presents literature exemplary cases from respiratory muscle training, strength training in endurance athletes, and exercise-based physical therapy. Moreover, the contextual role of TL in different populations and possible solutions regarding study design are discussed. We conclude that the considerations of TL assessment in research design may be beneficial for comparative experimental studies, crossover, and counterbalanced trials in sports sciences and physical therapy. By acknowledging and reconciling TL differences, researchers can elevate the relevance of their findings. Consequently, the effects of an intervention can be attributed to the type of intervention rather than confounded by differences in TL between groups.

## The role of training load for study design

Originally proposed by Banister et al. (1975), training load (TL) is a widespread concept based on the assumption that the dose and magnitude of training stimulus can be measured (Passfield et al. 2022). The term load is analogous to dose, referring to quantity or amount, whilst also accounting for the demand placed on the body (Impellizzeri et al. 2023). In sports and health sciences, TL refers to the cumulative amount of stress placed on an individual during exercise and competition, encompassing both the external workload (i.e., quantity of the work done by the athlete such as distance covered or weight lifted) and the internal response (i.e., quantity of the response to the stimulus such as heart rate (HR) or perceived exertion) (Borresen and Lambert 2009; Bourdon et al. 2017), and can be used to compare different loading modalities and strategies in exercise interventions (Dhahbi et al. 2024). For years, the complexity of TL has captivated the attention of sports and health practitioners, playing an important role in training prescription, injury mitigation, and fatigue management (Bourdon et al. 2017). As methodologies for quantifying TL continue to evolve, TL remains an important consideration for research in sports and physical therapy.

In exercise settings, various approaches have been employed to estimate TL, such as questionnaires, GPS tracking, physiological monitoring, and direct observation (Borresen and Lambert 2009; Bourdon et al. 2017), with such methods often used in combination to provide a comprehensive assessment of both the external workload and psychophysiological response. Metrics integrating different variables associated with TL include session-RPE (sRPE) and Training Impulse (TRIMP). The sRPE method combines the athlete’s rating of perceived exertion (RPE) with the duration of the training session, providing a simple yet effective measure of TL (Foster et al. 2001). sRPE has been successfully applied in team sports, endurance training (ET), and strength training (ST), and is considered a “standalone” method for TL monitoring (Sweet et al. 2004; Haddad et al. 2017). TRIMP, on the other hand, is an HR-based model that integrates exercise duration and HR response, often weighted by exercise intensity zones, to estimate the overall physiological stress of a session (Halson 2014). The TRIMP method has also been adapted for use in ST by substituting HR indices with the percentage of one-repetition maximum, and replacing exercise duration with the number of lifts performed (Busso et al. 1990). TRIMP has been recommended as a valid tool for TL monitoring as it consolidates both internal and external loads of ET and ST sessions into a single metric (Cejuela and Esteve-Lanao 2011; Martorelli et al. 2021). Alternative methods to quantify ET include the estimation of energy expenditure using metabolic equivalents of task (METs), which are used to standardize workload by converting different activities into a comparable unit (Mendes et al. 2018; Dhahbi et al. 2024). Since mechanical energy expenditure, relevant for strength and sprint training, can be measured using force plates, accelerometers, and linear position transducers, converting that across modalities using METs may also enable standardization of TL across different exercise modalities (Dhahbi et al. 2024). Furthermore, heart rate variability (HRV) can also be an effective tool for TL monitoring (Saboul et al. 2016) as a non-invasive and sensitive marker of autonomic nervous system activity that provides feedback on recovery status, stress levels, and readiness to train. HRV-derived metrics have already been successfully applied to TL assessment across endurance, strength, and skill-oriented activities (Kiviniemi et al. 2007; De Oliveira et al. 2019; Jin et al. 2022).

TL assessment and management are important because exercise-induced physiological responses depend on the dose of applied exercise stimulus. However, often studies are based on interventions resulting in different TL between the groups, possibly influencing the research outcomes. This may be especially relevant for research investigating performance in highly-trained athletes, where small differences usually determine success (Currell and Jeukendrup 2008). The core issue lies in potentially overseeing TL differences when assessing the efficacy of training or therapeutic interventions. Many studies compare groups exposed to different TL without recognizing this crucial factor. A common scenario is when one group engages in demanding supplementary activities while the control group does not, with limited recognition of the added TL experienced by the intervention group (Hauer et al. 2002; Millet et al. 2002; Spurrs et al. 2003; Holm et al. 2004; Beattie et al. 2017). Although such research design plays a necessary role in exploratory research, it exhibits limitations in studies examining effectiveness mediators, and crossover or counterbalanced trials. For instance, when a study aims to analyse whether additional ST is beneficial for endurance athletes, the difference in TL between the investigated groups may not be problematic. However, if the aim is to explore the general effectiveness of ST on endurance performance, TL differences between groups (ET only vs ET and ST) remain an important consideration. In such a scenario, adding ST would increase TL in the intervention group, thus endurance TL may need to be reduced by the same amount to maintain a matched TL between groups. If research seeks to isolate and analyse the specific effects of the investigated variable, equating TL seems important to minimize bias and possible confounders’ influence. Otherwise, it is not possible to differentiate whether the observed effects are associated with differences in TL or with the intervention (e.g., training, recovery, or nutrition strategy). The decision tree presented in Table 1 suggests a structured approach to determine the necessity of TL matching in experimental studies, based on design type, practical constraints, research aims, and outcome sensitivity.

**Table 1:** Decision tree for determining training load matching in experimental studies

1. Does the study compare two or more training interventions?		
	Yes	No
Example	Comparing resistance training vs plyometric training	Pre-post study assessing effects of a 6-week cycling program intervention
Action	Go to the next step	TL matching is not applicable
2. Is the study using a crossover design, and does each participant complete more than one intervention?		
	Yes	No
Example	Participants complete high-intensity interval and moderate-intensity continuous training in randomized order, with a washout period	Different groups perform either high-intensity interval training or moderate-intensity continuous training in a controlled trial design
Action	Matching TL would typically recommended to minimize carryover and order effects	Go to the next step
3. Are there contextual constraints that would make differences in TL between interventions impractical or unfeasible?		
	Yes	No
Example	Students exercise using different modalities during fixed-duration 45-min task	Elite cyclists train in a controlled setting with flexible programming and no major resource constraints
Action	Matching TL is recommended to maintain ecological validity within the real-world setting	Go to the next step
4. Is the primary aim to isolate the effect of the training type rather than the dose?		
	Yes	No
Example	Investigating if eccentric vs concentric strength training leads to different hypertrophy outcomes	Comparing low-load vs high-load strength training volume for hypertrophy
Action	Matching TL is recommended to ensure that observed effects are due to the training prescription and not differences in TL	TL matching may not be necessary if the aim is to compare real-world programs as they are typically implemented
5. Are the outcome variables highly sensitive to differences in TL?		
	Yes	No
Example	Comparing changes in physiological responses following two conditioning protocols	Comparing motivation, enjoyment, and exercise adherence rates between dance-based and treadmill walking interventions
Action	Matching TL may help reduce variability and improve the ecological validity of the results	Matching TL becomes less critical, but it should still be considered during the formulation of the study approach and when analysing the outcomes

Both ecological validity and applied practice are essential to balance scientific rigor with real-world relevance when considering TL in study design. Physical and mental resources are limited, and selecting the most effective exercise program whilst considering opportunity costs is crucial (Buchanan 1991). In an athletic context, the opportunity cost refers to the benefits sacrificed when choosing a training, recovery, or strategy over another. The concept highlights the trade-offs athletes and coaches face when allocating time, effort, and resources. Thus, although many intervention strategies may improve performance, only the most effective are typically implemented. Therefore, to assess the “return on investment”, potential benefits of a training intervention may be better quantified in the context of “invested TL”. Considering “invested TL” is equally critical in physical therapy (PT), as patients often have limited exercise tolerance and may exhibit larger stress physiological responses compared to young and healthy populations, even at low or moderate load (Athanasiou et al. 2023). Therefore, establishing an appropriate exercise dose and intervention type is important for PT intervention research. Importantly, while statistical methods can adjust for TL differences to some extent, they cannot fully account for unmeasured confounders or the complex nonlinear effects of training over time. Additionally, TL is often influenced by individual adaptations and external factors that may not be adequately captured in statistical models. As a result, controlling TL by study design remains the most reliable approach to minimizing bias and making valid causal inferences in comparative studies, investigating the isolated effects of applied interventions. Subsequently, this viewpoint aims to discuss three literature-based exercise interventions – respiratory muscle training (RMT), ST in endurance athletes, and exercise interventions in PT – to elucidate the contextual role of TL among different populations. Finally, considerations for study design and further research are presented.

## Example 1: respiratory muscle training

RMT has been demonstrated to be an effective training tool in healthy and athletic populations (Illi et al. 2012; HajGhanbari et al. 2013; Kowalski et al. 2024), typically improving maximal inspiratory and expiratory pressures (Sapienza et al. 2002; Kowalski et al. 2024), aerobic performance (Illi et al. 2012), and respiratory muscle function (González-Montesinos et al. 2012; Sales et al. 2016). Furthermore, positive effects have been observed on blood lactate accumulation and perceived exertion during exercise (HajGhanbari et al. 2013). In well-controlled studies assessing RMT effects, the experimental group undergoes an RMT program while the control group usually engages in sham training (McConnell and Romer 2004). Systematic reviews on RMT’s influence on exercise performance reported a time trial improvement of 2% after RMT compared to controls, and no influence on maximum oxygen uptake (V̇O₂max) (Illi et al. 2012; HajGhanbari et al. 2013).

RMT has re-gained significant attention following the COVID-19 pandemic (Morgan et al. 2024; Vranić et al. 2024), with studies advancing our understanding of RMT-associated fatigue, muscle damage, stress, and TL (Briskey et al. 2020; Kowalski et al. 2023; Iqbal et al. 2023), which may facilitate TL-matching future investigations. For example, TL associated with RMT has been recently reported to account for 1.6% to 6.6% of the total TL (assessed as sRPE) of well-trained triathletes (Kowalski et al. 2023). Interestingly, to the best of our knowledge, despite decades of research on RMT in athletes, a comparison between RMT and increased sport-specific training has not been investigated yet. Noteworthy, TL associated with RMT interventions may be estimated with the sRPE metrics (Doria et al. 2018; Kowalski et al. 2023), potentially allowing for RMT’s effectiveness compared to other training strategies with improved methodological rigor. This seems especially important for elite athletes, given that despite decades of exploration the effect of RMT on such a population remains debated (McConnell 2012; Patel et al. 2012).

## Example 2: strength training in endurance athletes

It is well known that ST enhances performance in endurance athletes, with several reviews summarising the findings of the past 30 years (Rønnestad and Mujika 2014; Blagrove et al. 2018; Llanos-Lagos et al. 2024). Physiological adaptations from ST are suggested to include increased musculotendinous stiffness, reduced muscle fiber recruitment at the same absolute speed/load, enhanced motor unit synchronization, and an augmented rate of force development (Mujika et al. 2016; Blagrove et al. 2018; Trowell et al. 2020). Consequently, ST may enhance endurance performance via improved exercise economy, velocity/power output at V̇O₂max, and anaerobic characteristics (Mikkola et al. 2007; Yamamoto et al. 2008; Beattie et al. 2014; Feuerbacher and Schumann 2023; Zanini et al., 2025)

It is important to note that, while ST generally improves endurance performance, most studies introduce ST in addition to ET, while the control group continues their regular ET (Johnson et al. 1997; Millet et al. 2002; Beattie et al. 2017), and the differential TL may influence study outcomes. A recent review by Llanos-Lagos et al. (2024) examining ST interventions in ~ 900 athletes across 31 studies found that in most investigations ST was added to the regular ET training in the intervention group (Llanos-Lagos et al. 2024), implying an unmatched TL change between groups. Though a few investigations have also shown the positive influence of ST in endurance athletes when ET was reduced to maintain similar training volumes between the experimental and control groups (Paavolainen et al. 1999; Mikkola et al. 2007), more research in this area is warranted. These adjustments partially address the issue and reduce discrepancies in TL, although it is likely that TL differences between groups are still present and may impact the study outcome.

TL matching may depend on the experimental design, and such an approach seems reasonable in applied studies investigating ST interventions for endurance performance enhancement. This may be particularly relevant for recreational athletes who usually have limited time to train, hence increasing the TL in an intervention group without adjustments to controls may limit the results’ translation to real-world situations. A potential approach for future studies focusing on applied performance may therefore be to equate the additive TL in the intervention group to an equivalent ET in the control population, based on measures of TL applicable to ST and ET such as TRIMP or sRPE. It is also worth acknowledging that some studies may not benefit from TL matching, especially in studies primarily focusing on physiological adaptations rather than applied performance outcomes.

## Example 3: exercise interventions in physical therapy

Physical activity in PT is crucial, as lack of exercise in patients is often associated with additional health issues such as muscle weakness and lowered exercise capacity (Decramer et al. 1997). For example, patients with cardiorespiratory disorders experience excessive symptoms and reduced work capacity due to a combination of factors, including not only difficulties with breathing, gas exchange, and circulation, but also underlying muscular weakness (Ortega et al. 2002). Therefore, medical societies consider physical training with different types of exercise a necessary component of PT. However, no optimal approach focusing on a singular activity is generally recommended, as the effectiveness of the therapy depends on disease and population, among other factors (Cauza et al. 2005; Daabis et al. 2017; Berry et al. 2018). Consequently, comparative studies investigating different exercise interventions are often implemented to optimize the effectiveness and feasibility of the interventions.

A popular study design in PT is to compare different exercise prescriptions, such as programs based on different session frequency, volume-to-intensity ratio, or a distinctive focus on ET and ST (Wisløff et al. 2007; Heyn et al. 2008; Daabis et al. 2017; Makarewicz et al. 2022). Importantly, most of these studies have not accounted for TL matching in their design. For instance, in a systematic review evaluating the effects of exercise on fatigue in individuals with chronic obstructive pulmonary disease (COPD), Li et al. (2019) reported that only 3 out of 17 studies demonstrated consistency in workload prescription between the ET, ST and the combined ET + ST training groups (Li et al. 2019). Similarly, a systematic review comparing ST and ET in COPD by Iepsen et al. (2015) reported that only 6 out of 11 studies presented the TLs for training interventions (Iepsen et al. 2015), further highlighting how TL may often be overlooked in PT-related studies. Furthermore, in another recent review investigating the effects of ET, ST, and combined training in overweight and obese adults, in 11 out of 24 studies the session’s duration or intensity was not described (Makarewicz et al. 2022), making a TL comparison between conditions impossible.

Although comparing TL between different types of intervention constitutes a challenge and is rarely applied, an attempt to match TL between conditions is often possible with TL metrics such as sRPE, TRIMP, or energy expenditure expressed with METs. For endurance-oriented therapies, the approach have been applied by Wisløff et al. (2007), where moderate and high-intensity training programs were compared in terms of their effects on cardiovascular function and prognosis in patients with post-infarction heart failure. The intervention protocols were designed to be isocaloric, ensuring that the total energy expenditure was equivalent across groups (Wisløff et al. 2007), which allowed for an isolation of exercise intensity effects. METs-based energy expenditure has also been used to compare TL between very distinct activities by Wang et al. (2025) that addressed optimal dose and type of exercise in Parkinson's disease across a range of exercise modalities (including dance, Tai Chi, and sensory training) (Wang et al. 2025). Given the above evidence, it seems important to report interventions’ TL for the replicability and robustness of studies in PT. Thus, we suggest including TL assessment and matching in research comparing different interventions, using metrics such as sRPE, TRIMP, and energy expenditure expressed with METs. Consequently, the effects of the intervention can be attributed to the type of intervention itself, rather than to differences in TL.

## Between-populations differences in training load considerations

**Fig. 1:**
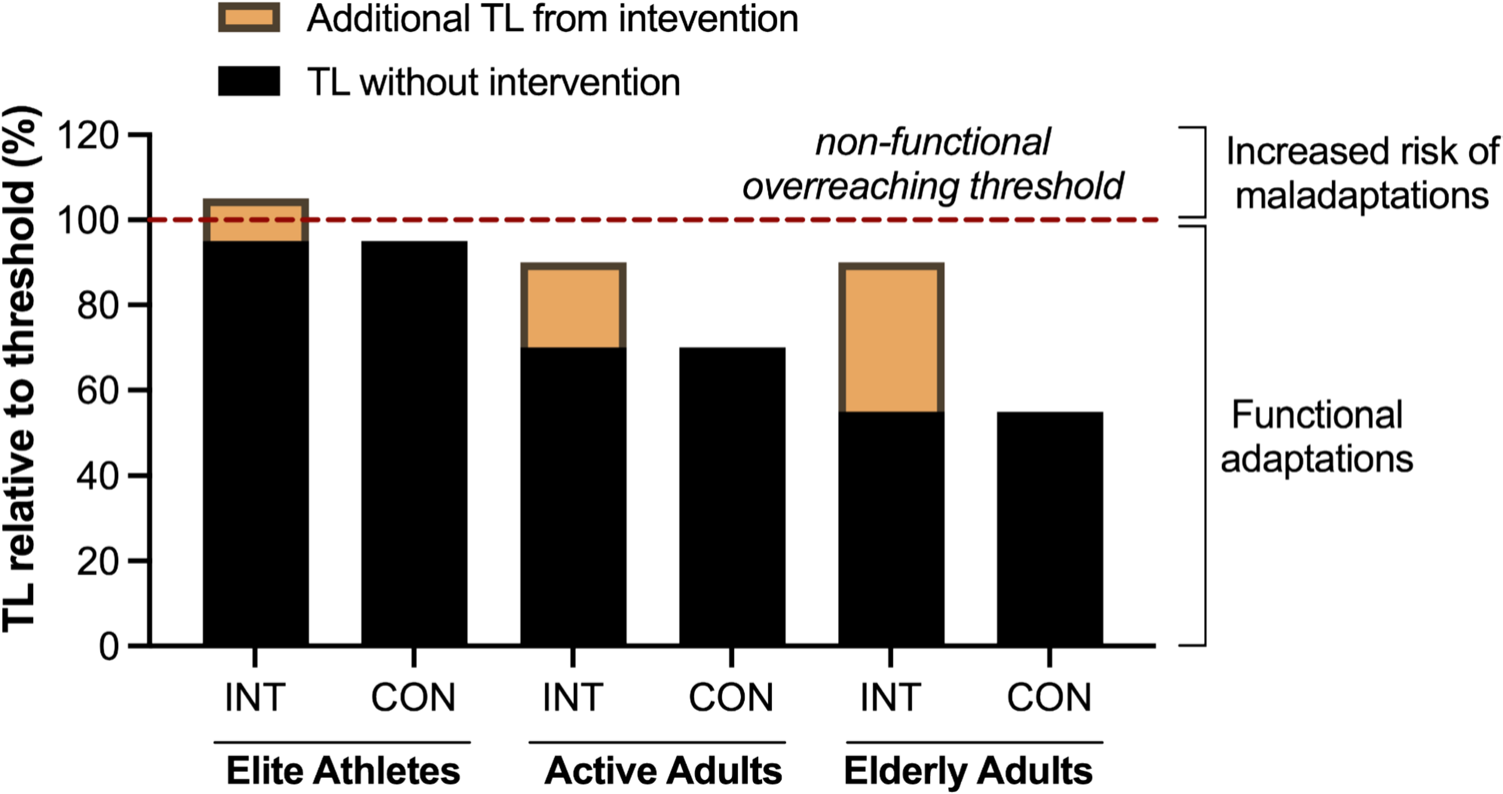
Effects of several weeks of additional training load (TL) in intervention groups (INT) across different populations compared to controls (CON), represented as the TL percentage relative to an individual hypothetical non-functional overreaching threshold. The additional TL may contribute to overreaching in populations already training close to their adaptive capacity (i.e., elite athletes) — but increase the amount of productive training in populations further from their upper-limit capacity. Furthermore, an increased external load is likely to have a larger influence on the internal load in less fit populations, as represented in the figure. The presented overreaching threshold is only illustrated as a mental model, as overreaching and overtraining are understood as a continuum spanning from disturbances in adaptation to significant maladaptation (Meeusen et al. 2013), and not as a specific and defined boundary

It is important to highlight that as elite athletes operate near their physiological limits, even a small increase in TL may lead to overreaching or overtraining. While overreaching is critical for eliciting post-training adaptations, it requires precise and timely management, which can differentiate functional and non-functional overreaching (fatigue lasting weeks to months, associated with maladaptation) (Schwellnus et al. 2016). Consequently, interventions leading to an increased TL lasting weeks or months may at times be counterproductive, as performance could decrease compared to controls with lower TL. On the other hand, interventions with a TL increase in recreationally active or elderly participants typically elicit larger positive changes, as these populations operate further from their individual overtraining/overreaching threshold, which is unlikely to be overcome by the increased TL from an intervention. These concepts are represented in Fig. 1.

As shown in Fig. 1, similar objective TL may correspond to different subjective TL. For example, 50 min of running at an intensity corresponding to 70% of V̇O₂max may constitute an active recovery session for a well-trained runner and a demanding effort for an untrained subject. Therefore, similar exercise protocols may provide a stronger stimulus and elicit larger adaptations in untrained, compared to trained populations. For instance, Yamamoto et al. (2008) reported that most studies investigating the influence of ST on running performance included untrained subjects, which may have played a significant role in the improvements in running performance or economy (Yamamoto et al. 2008). In well-trained runners, ST would typically constitute a relatively smaller load, and therefore the associated performance enhancement may be smaller. Additionally, in these studies, the untrained subjects usually run 2–4 times per week, therefore the additional fatigue from ST may not have particularly influenced their usual ET. On the other hand, well-trained and elite runners may exercise up to 15 times per week, and additional fatigue coming from ST sessions may have an impact on subsequent sessions (Van Hooren et al. 2024). Therefore, in this example, both the stimulus’s magnitude and the ST-associated trade-offs may affect adaptations to ST in ET. These observations underline the limited transferability of training intervention effects between populations of distinctive fitness status, training routines, or health conditions.

## Future directions

In this viewpoint, we have highlighted the role of TL in comparative experimental research. Rather than disregarding differences in TL, studies could aim to equate TL across experimental and control groups through tailored interventions. In particular, a TL matching may be beneficial in crossover designs, when practical constraints limit what participants can do, or when the primary aim is to compare different interventions. Such an approach allows for assessing the effects of interventions while mitigating the effect of TL on study outcomes. In contrast, matching TL may be less useful when evaluating real-world training programs or outcomes less sensitive to TL variations, like enjoyment or adherence to exercise. Differences in TL between the groups should also be acknowledged as a study limitation in interventions where TL matching is advisable.

Practical strategies for matching TL in intervention studies may be guided by literature reviews or exploratory studies. One approach could involve estimating the intervention group's TL first, and subsequently matching it within the control group's program using TL metrics such as sRPE or TRIMP, which can be calculated across different exercise modalities (Sweet et al. 2004; Haddad et al. 2017). It must also be acknowledged that estimating TL for training prescriptions is more challenging than measuring TL as part of the monitoring process. Such difficulty directly affects the ability to prospectively align TL across various interventions when designing a study. In this context, relying on external rather than internal variables currently remains easier and more feasible. As a result, studies may tend to over-rely on external variables for TL matching between interventions, although dissociation between external and internal load depends on the magnitude of fatigue and exhibits high intra-subject variability (Halson 2014). Consequently, leveraging technological advancements to assess external and internal workload measures and account for individual responses to training may enhance the accuracy of TL quantification (Borresen and Lambert 2009; Mujika 2017). The work of Hebisz and Hebisz (2024) provides a useful starting point in this area, with TL continuously adjusted based on the morning HRV measure during ET intervention (Hebisz and Hebisz 2024). A similar approach has also been used by other studies on ET and ST and may be an avenue for future studies to match and adjust TL (Kiviniemi et al. 2007; Javaloyes et al. 2019; De Oliveira et al. 2019).

Importantly, standardizing training interventions for TL will enhance research quality only if the methods used to measure TL are valid, and the existing TL assessment methods should be applied contextually to account for their shortcomings (Bourdon et al. 2017). Therefore, a potential avenue for future research might also include a retrospective TL assessment relying on parameters linked to the psycho-physiological state of the athletes, which mostly relies upon stress responses to the performed training. This might include resting HR, HRV, or internal-to-external load ratio analysis, as all provide insight into athletes’ fatigue and training status (Bourdon et al. 2017; Altini and Plews 2021).

In conclusion, incorporating matched TL is advisable in multiple study designs that are commonly applied in sports science and physical therapy research, and aligning TL differences may enhance the rigor and applicability of research findings. Whether different TL between groups constitutes a study limitation depends on the primary aims and research questions. If necessary, specific metrics like sRPE, TRIMP, or energy expenditure expressed with METs may be used to quantify and match TL between investigated groups. Otherwise, a lack of matched TL should be recognized and highlighted as a limitation.
